# Ecological drivers for poultry farms predisposed to highly pathogenic avian influenza virus infection during the initial phase of the six outbreaks between 2010–2021: a nationwide study in South Korea

**DOI:** 10.3389/fvets.2023.1278852

**Published:** 2023-12-07

**Authors:** Kyung-Duk Min, Dae-sung Yoo

**Affiliations:** ^1^College of Veterinary Medicine, Chungbuk National University, Cheongju, Republic of Korea; ^2^College of Veterinary Medicine, Chonnam National University, Gwangju, Republic of Korea

**Keywords:** highly pathogenic avian influenza, veterinary epidemiology, conservation epidemiology, predator species richness, environmental drivers

## Abstract

**Background:**

Highly pathogenic avian influenza (HPAI) has caused substantial economic losses worldwide. An understanding of the environmental drivers that contribute to spillover transmission from wild birds to poultry farms is important for predicting areas at risk of introduction and developing risk-based surveillance strategies. We conducted an epidemiological study using data from six HPAI outbreak events in South Korea.

**Materials and methods:**

An aggregate-level study design was implemented using third-level administrative units in South Korea. Only regions with high natural reservoir suitability were included. The incidence of HPAI at chicken and duck farms during the initial phase (30 and 45 days after the first case) of each outbreak event was used as the outcome variable, assuming that cross-species transmission from wild birds was the dominant exposure leading to infection. Candidate environmental drivers were meteorological factors, including temperature, precipitation, humidity, and altitude, as well as the proportion of protected area, farm density, deforestation level, and predator species richness. Logistic regression models were implemented; conditional autoregression models were used in cases of spatial autocorrelation of residuals.

**Results:**

Lower temperature, higher farm density, and lower predator species richness were significantly associated with a higher risk of HPAI infection on chicken farms. Lower temperature, higher proportion of protected area, and lower predator species richness were significantly associated with a higher risk of HPAI infection on duck farms.

**Conclusion:**

The predicted dominant transmission routes on chicken and duck farms were horizontal and spillover, respectively. These results reveal a potential protective effect of predator species richness against HPAI outbreaks. Further studies are required to confirm a causal relationship.

## 1 Introduction

The avian influenza virus, belonging to the Orthomyxoviridae family, is primarily categorized into highly pathogenic avian influenza virus (HPAIv) and low pathogenic avian influenza virus (LPAIv), primarily based on its level of pathogenicity in chickens, as per the guidelines provided by the World Animal Health Organization (OIE). Chickens or turkeys that come into contact with HPAIv typically manifest acute systemic symptoms, ultimately resulting in mortality (1). For example, chickens and turkeys infected with HPAIV develop acute respiratory symptoms that result in nearly 100% mortality. Additionally, avian influenza viruses (especially H5N1 and H7N9) are classified as zoonotic agents that cause high mortality in humans (2). For example, 36% mortality in humans was reported during an avian influenza H7N9 outbreak in China (3), which led to concern among the general public and caused health authorities to implement systematic control and prevention strategies (4).

South Korea experienced several HPAI epidemics between 2003 and 2020, during which three HPAIV subtypes (H5N1, H5N8, and H5N6) spread across the county. The H5N1 subtype of HPAIV affected poultry (mainly chicken) farms in South Korea from 2003 to 2011: 19 cases in 2003, 7 cases in 2006–2007, 33 cases in 2008, 53 cases in 2010, and 53 cases in 2011. Overall, 392 cases occurred during the HPAIV H5N8 epidemic from 2014 to 2015, particularly on domestic duck farms (75.8%), and primarily in three waves lasting 195, 268, and 64 days (5). Moreover, the H5N6 subtype affected 343 poultry farms during the 2016–2017 epidemic (5). Furthermore, in the year 2017, a total of 76 cases of H5N8 and 22 cases of H5N6 were officially identified within poultry farms. Following a series of consecutive HPAI outbreaks, two years later, South Korea experienced 108 cases of H5N8 in poultry farms, along with an additional 274 cases of H5N8 confirmed in wild birds during the 2020–2021 epidemic. Since the onset of 2020, there has been a recurring annual reintroduction of the HPAI virus, leading to the identification of 47 cases of H5N1 during the 2021–2022 epidemic. More recently, in the period spanning 2022–2023, a total of 75 cases of H5N1 were confirmed within poultry farms (6).

National biosecurity management and surveillance systems for HPAI were enhanced and reorganized after the massive 2016–2017 H5N6 epidemic. However, poultry farms continued to be affected after HPAIV was identified in wild birds, despite radical and stringent regulations by animal health authorities (7).

Recently, the subtypes that caused the previous epidemics (H5N8 and H5N1) have reemerged nationwide and caused further damage to poultry production and the supply chain (8). The impact of HPAIV emergence on the poultry industry has increased, mainly because of the greater susceptibility and presence of subclinical infections in domestic duck species, which hinder early virus detection and reporting, thus permitting lateral transmission to poultry farms.

Since 2003, HPAI outbreaks have impacted nearly all regions of the country. Nevertheless, the majority of these outbreaks have been concentrated on poultry farms situated along the southwestern coast of the Korean peninsula (5, 9, 10). This area is a wintering site for migratory birds, and several HPAIVs have been identified among wild bird populations in the area (11, 12). Regardless of HPAIV subtype, poultry farms infected during the initial phase of the epidemic were often located in specific regions, near sites of confirmed HPAI infection in wild birds (13). For example, both the H5N8 epidemic in 2014 and the H5N6 epidemic in 2016 initiated from a limited number of domestic duck farms in Jeollabuk-do, situated in close proximity to areas where HPAI viruses had been detected in wild birds (13). These geographical patterns of different epidemics suggest that there is a geographical preference for HPAIV introduction into poultry farms through diverse factors, such as wild bird migration (14). Therefore, it is important to identify factors contributing to the location of the early stages of an epidemic; early detection and prompt response can help to identify locations with a greater likelihood of HPAI infection, facilitate biosecurity resource allocation, and support prevention strategies in regions with higher HPAI risk.

The results of recent studies suggest that ecological diversity is linked to infectious disease susceptibility (15–17). For example, one study showed that species richness was positively associated with outbreaks of Zika virus infection. This finding prompted us to explore whether farms or regions highly connected to surrounding ecosystems are likely to be involved in the initial phase of an outbreak; this relationship may be represented by ecosystem connection strength, referred to as the intensity or magnitude of ecological relationships that exist among various species, populations, or abiotic factors within an ecosystem, species richness, or species evenness, or another diversity index (18).

Thus far, there have been few studies regarding factors associated with the initial phase of HPAI outbreaks in poultry farms. Here, we examined connections between surrounding ecosystems and poultry farms during the initial phase of HPAI outbreaks. We performed spatial analyses using six HPAI outbreaks between 2010–2021 (the outbreaks in 2010–2011, 2014–2016, 2016–2017, 2017, 2017–2018, and 2020–2021), with the goal of identifying associations between ecological factors and early infection.

## 2 Materials and methods

### 2.1 Study design

An aggregate-level study design was utilized to estimate associations between HPAI outbreaks on poultry farms and environmental drivers. The study unit was the neighborhood, which is the third-level administrative unit in South Korea. There are 17,254 neighborhoods, with an average size of 5.80 km^2^. Additionally, a case–control design was implemented. Considering that HPAI infection occurs by spillover (transmission from wild birds to poultry farms; the focus of this study), as well as horizontal transmission (transmission between poultry farms by workers and vehicles), inclusion criteria for case and control neighborhoods were carefully constructed.

**Figure 1:**
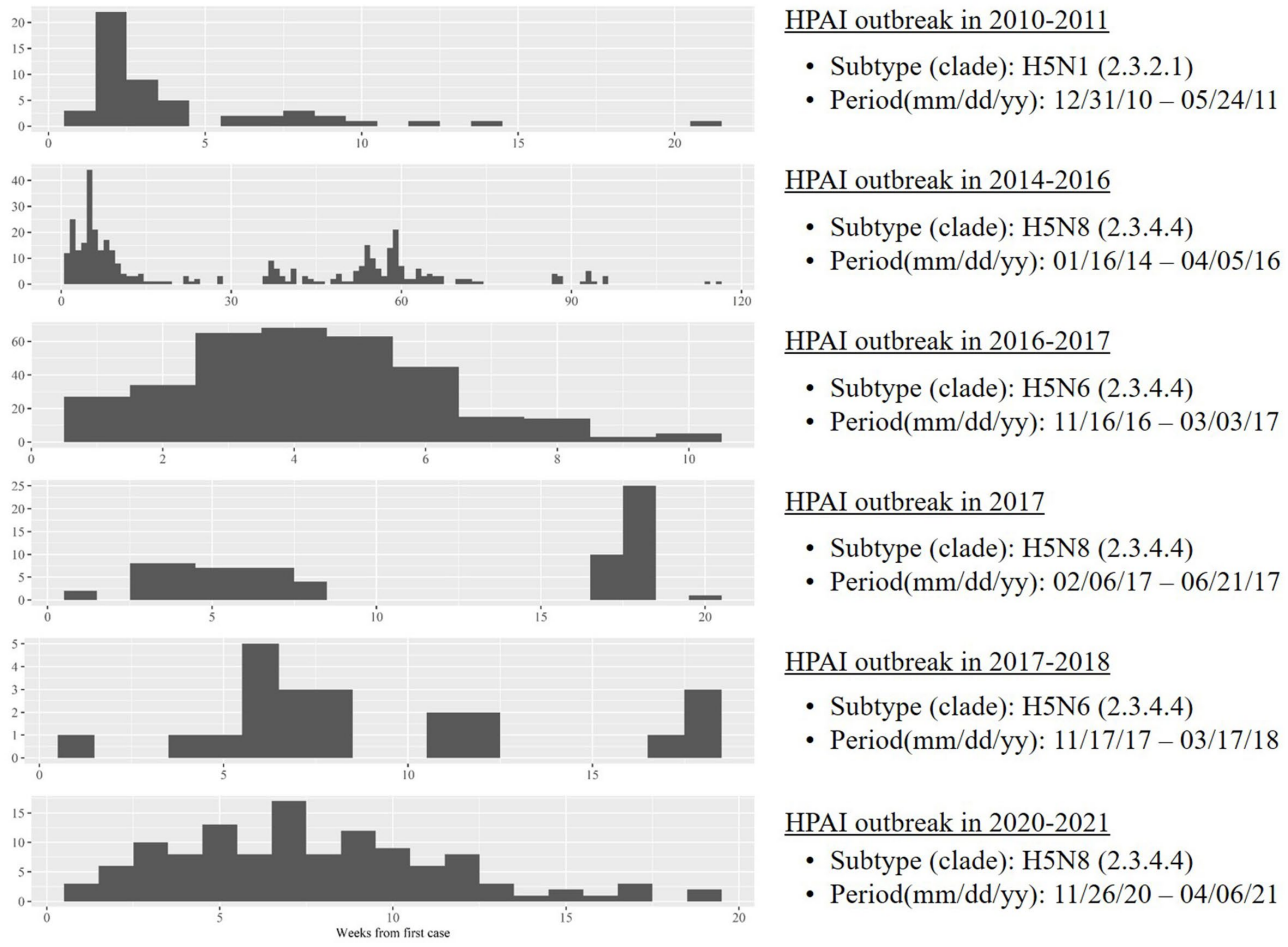
Temporal trends in highly pathogenic avian influenza on poultry farms during six outbreaks between 2010 and 2021 in South Korea.

Case neighborhoods were operationally defined as regions containing farms with HPAI infection by spillover from wild birds, according to two inclusion criteria: regions containing farms with HPAI infection during the initial phase of an outbreak, and regions that are highly suitable as HPAI reservoirs. The first criterion reflects the previous finding that a major source of infection during the initial phase of an HPAI outbreak is spillover from wild birds; during later phases, horizontal transmission is the major source (19). Because there are no clear criteria for the initial phase of an HPAI outbreak, two time periods were examined to ensure robust analysis: 30 days and 45 days. Neighborhoods that satisfied both criteria were selected as case regions. Control neighborhoods were operationally defined as regions that are highly suitable as HPAI reservoirs but contain no HPAI-infected farms. Six outbreaks were reported in South Korea during the study period (20) (Figure 1); the analysis period comprised the first 30 and 45 days since the first case in each outbreak (Supplementary Figures S1–S7).

**Figure 2:**
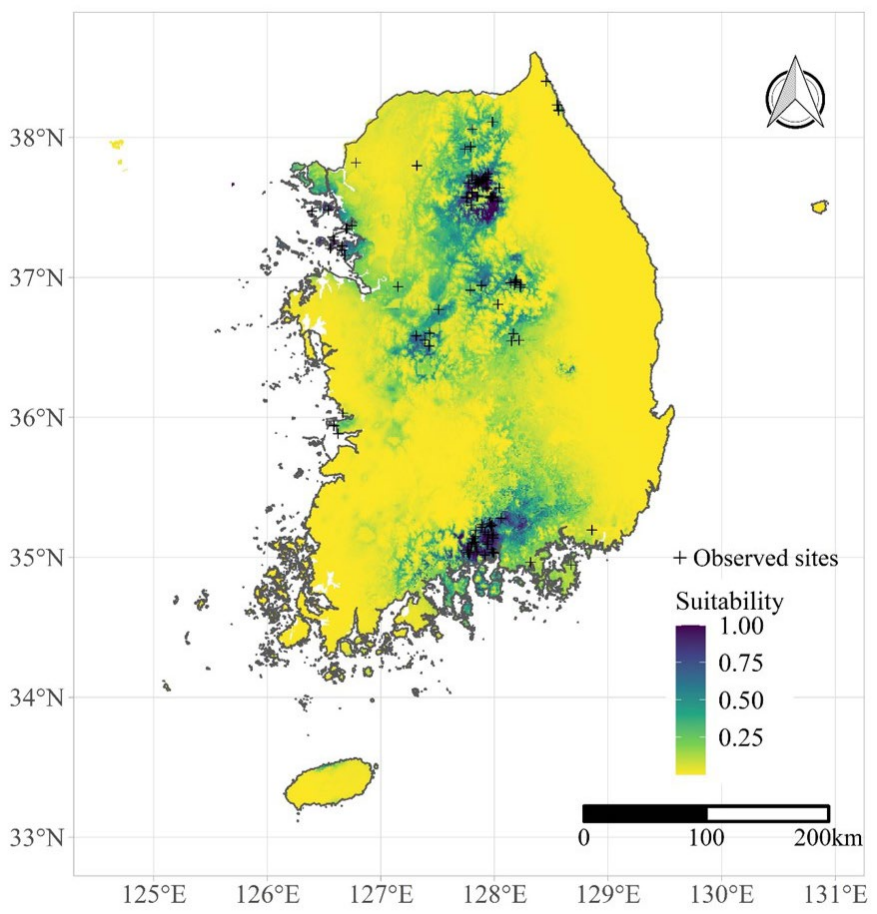
Suitability of *Anas poecilorhyncha* predicted by the maxent model.

HPAI reservoir suitability was examined using the suitability of *Anas poecilorhyncha*. Suitability was estimated by the maxent model in R v.4.3.1 (21). The maxent function in the *dismo* package (22) was employed, and species occurrence data were obtained from the National Ecosystem Survey (23) conducted by the National Institute of Ecology between 2006 and 2013. The maxent model demonstrated good performance in terms of data fitting, with area under the curve values >0.9. The suitability of *A. poecilorhyncha* predicted by the maxent model is illustrated in Figure 2. Although *Anas platyrhynchos* is also a dominant reservoir species in South Korea, the geographical variation of their occurrence data was not enough to develop a maxent model (Supplementary Figure S8). Therefore, we only used suitability of *A. poecilorhyncha* as reservoir suitability variable.

### 2.2 Data acquisition and preprocessing

Eight environmental variables, including meteorological factors, the proportion of protected area, deforestation level, altitude, poultry farm density, and predator species richness were used as explanatory variables in this study (Table 1).

**Table 1:** Data utilized in this study.

Variables	Spatial resolution	Temporal resolution	Data source
Locations of predators	District-level	NA	NES
Mean temperature	Point-level	Monthly	ASOS
Mean humidity	Point-level	Monthly	ASOS
Mean precipitation	Point-level	Monthly	ASOS
Proportion of protected area	100-m pixel	NA	KNGII
Deforestation	30-m pixel	Yearly	GFC
Altitude	90-m pixel	NA	SRTM
Poultry farm location	Point-level	NA	MOIS

Data sources for predator location, meteorological factors, protected area, deforestation, elevation, and farm locations were the National Ecosystem Survey (NES), Automatic Synoptic Observation System (ASOS), Korea National Geographic Information Institute (KNGII), Global Forest Change (GFC), Shuttle Radar Topography Mission (SRTM), and Ministry of the Interior and Safety (MOIS).

Average temperature, relative humidity, and precipitation were used as explanatory meteorological variables. The time for calculating the average value was the first 2 months of each outbreak: December 2010, January 2011, January 2014, February 2014, November 2016, December 2016, November 2017, December 2017, November 2020, and December 2020. Raw data were obtained from the automatic synoptic observation system (24), which provided monthly mean data for each monitoring site, as well as the geographic locations of the monitoring sites (i.e., coordinates). Representative values of meteorological factors for each neighborhood were estimated by ordinary kriging, a spatial interpolation method.

The proportion of protected area for each neighborhood was estimated using the ecological natural index, an ordinal variable consisting of three categories, which was designed to classify regions according to the abundances of vegetation abundance and endangered wildlife species for land use and development. The ecological natural index across South Korea was extracted in a raster file format with 100-m resolution from the eco-natural map constructed by the Korea National Geographic Information Institute in 2016. The first category of the variable represents regions with high abundances of vegetation and wildlife species at each geographic location, which are needed for conservation and recovery of the natural environment. Since we only explicitly consider *A. poecilorhyncha* as a reservoir in study design, inclusion of protected area variable can address unmeasured factors regarding HPAI reservoirs. We estimated the proportion of protected area for each neighborhood using the proportion of the number of pixels representing the first category and the number of pixels for all categories.

The deforestation level for each neighborhood was estimated using data from Global Forest Change (25, 26). Raster-type spatial data with 30-m pixel resolution were acquired, and a variable representing deforestation events was available for each pixel. Specifically, the variable indicated whether there were deforestation events during each year between 2001 and 2020. In this study, the proportion of deforested area in each neighborhood was calculated as the sum of the number of pixels representing deforestation events between 2001 and 2020, divided by the number of pixels in the neighborhood.

The altitude of each neighborhood was obtained from raster-type data at 90-m resolution (27). After extraction of all altitude values for a given neighborhood, the average altitude was calculated.

The geographic locations of chicken and duck farms were obtained from the LOCAL DATA website (28), an open-source big data repository for building locations in South Korea operated by the Ministry of the Interior and Safety. Coordinates of operational chicken and duck farms are provided in the repository. The numbers of chicken and duck farms in each neighborhood were recorded. Subsequently, the numbers were divided by the spatial extent of each neighborhood to determine the densities of chicken and duck farms.

Predator species richness was defined as the number of predator species identified by the National Ecosystem Survey (23) between 2006 and 2013. We included the variable because higher richness of predators could reduce activity of the reservoirs which possibly reduces risk of spillover to poultry farms. Although the National Institute of Ecology provides point-level data for the occurrence of most wildlife species, only district-level (second administrative level in South Korea) occurrence data are available for predator species. Because most predator species are endangered in South Korea, disclosure of their location data is prohibited to protect against hunting. The occurrence data of six predator species (four avian and two mammalian) were used to estimate predator species richness: *Falco peregrinus, Accipiter gentilis, Buteo buteo, Haliaeetus albicilla, Prionailurus bengalensis, and Martes flavigula*.

The analysis involves data only in aggregate form and no personal information has been collected. Because we did not use any human data or human materials, ethics approval, consent to participate, and anonymization are not required.

### 2.3 Statistical analysis

Associations between environmental factors and HPAI presence during the initial phase of an outbreak were examined by multivariate logistic regression models with odds ratios (ORs) and 95% confidence intervals. Twelve logistic regression models were used in this study (six models each for chicken and duck farms). The inclusion criteria for the study units and the numbers of case and control regions for each model are shown in Supplementary Table S1. A descriptive analysis was conducted to summarize the features of environmental factors for case and control regions. For simplicity, the descriptive analysis was only conducted for model 1. Variance inflation factor values were estimated for explanatory variables to identify multicollinearity (29). Moran’s I-statistic and corresponding *p*-values were estimated for each model to examine spatial autocorrelation. If spatial autocorrelation was detected, conditional autoregressive models were implemented using the “CARBayes” package (30).

## 3 Results

Differences in environmental and geographical features between regions with HPAI cases during the initial phase of an outbreak (case regions) and regions without HPAI cases (control regions) are shown in Table 2, using the model 1 dataset. No significant differences in mean temperature, mean humidity, mean precipitation, or conservation level were observed for chicken farms. However, the deforestation level, altitude, and predator species richness were significantly higher in control regions; farm density was significantly higher in case regions. In contrast, mean temperature, mean humidity, the proportion of protected area, and predator species richness were significantly different on duck farms; differences in mean precipitation, deforestation level, altitude, and farm density were not statistically significant.

**Table 2:** Descriptive analysis of environmental factors in the study regions.

Variables	Regions with chicken farms			Regions with duck farms		
	Case (*N* = 14)	Control (*N* = 385)	*p*-value	Case (*N* = 20)	Control (*N* = 38)	*p*-value
Mean temperature	1.29 ± 1.5	1.26 ± 1.8	0.949	0.83 ± 0.2	1.60 ± 1.3	< 0.001
Mean humidity	63.15 ± 3.1	62.78 ± 2.8	0.669	64.27 ± 0.3	62.64 ± 1.9	< 0.001
Mean precipitation	23.44 ± 2.8	23.35 ± 3.5	0.908	21.67 ± 0.9	21.45 ± 2.4	0.618
Protected area	31.87 ± 30.3	16.03 ± 21.7	0.073	55.45 ± 34.3	29.87 ± 31.3	0.009
Deforestation	0.81 ± 2.3	3.62 ± 6.2	< 0.001	2.58 ± 6.9	4.12 ± 8.7	0.467
Altitude	75.01 ± 32.3	126.74 ± 81.4	< 0.001	88.56 ± 14.9	83.84 ± 37.9	0.502
Farm density	1.70 ± 1.8	0.42 ± 0.5	0.019	0.77 ± 1.2	0.35 ± 0.8	0.159
Predator species richness	2.79 ± 0.8	3.45 ± 1.1	0.009	2.70 ± 0.7	3.42 ± 1.0	0.002

An ecological study design was implemented, and the unit of analysis was the neighborhood. The analysis was stratified according to animal species (chicken and duck farms). Six models were established. Because the inclusion criteria for the case and control regions were different, the numbers of case and control regions differed for each model. The table shows descriptive results using the regions included in model 1.

**Figure 3:**
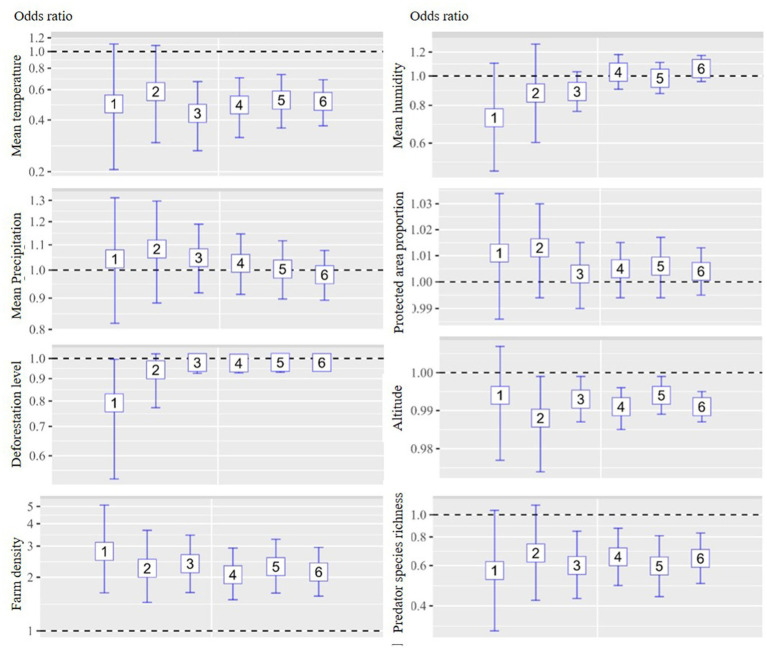
Associations between environmental drivers and the incidence of HPAI in chicken farms during the initial phase of an outbreak by six models. An ecological study design was implemented, and the unit of analysis was the neighborhood. The analysis was stratified according to animal species (chicken and duck farms). Multivariate logistic regression analysis was implemented for each model, and eight explanatory variables were included: mean temperature, precipitation, humidity, deforestation, protected area proportion, farm density, altitude, and predator species richness. Inclusion criteria for study regions were different by models. Regions with top 10%, 20%, and 30% reservoir suitability were included in model 1–2, 3–4, and 5–6, respectively. Operational definition of the initial phase of an outbreak were 30 days for model 1, 3, and 5 and 45 days for model 2, 4, and 6.

**Figure 4:**
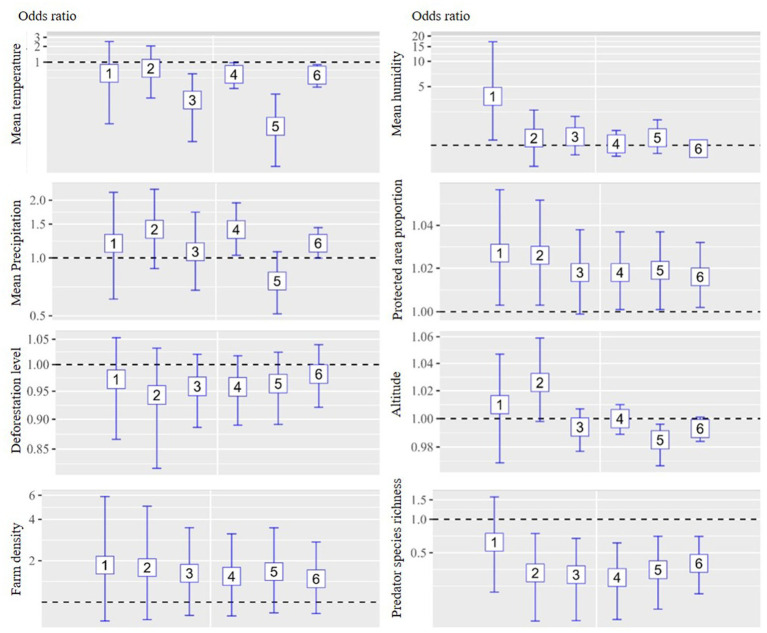
Associations between environmental drivers and the incidence of HPAI in duck farms during the initial phase of an outbreak by six models. An ecological study design was implemented, and the unit of analysis was the neighborhood. The analysis was stratified according to animal species (chicken and duck farms). Multivariate logistic regression analysis was implemented for each model, and eight explanatory variables were included: mean temperature, precipitation, humidity, deforestation, protected area proportion, farm density, altitude, and predator species richness. Regions with top 10%, 20%, and 30% reservoir suitability were included in model 1–2, 3–4, and 5–6, respectively. Operational definition of the initial phase of an outbreak were 30 days for model 1, 3, and 5 and 45 days for model 2, 4, and 6.

The multivariate analysis of the six models is depicted in Figures 3, 4 for chicken and duck farms, respectively. Mean temperature was negatively associated with HPAI on both chicken and duck farms. Significant associations were identified in models 3–6 for chicken farms; the respective ORs were 0.437, 0.489, 0.522, and 0.513. Significant associations were identified in models 3–6 for duck farms; the respective ORs were 0.188, 0.585, 0.059, and 0.565. However, the associations with mean humidity and precipitation were not statistically significant. The deforestation level was not statistically significant on chicken or duck farms. Farm density was positively associated with incidence on chicken farms (all models showed significant associations; the respective ORs for models 1–6 were 2.798, 2.249, 2.386, 2.068, 2.298, and 2.144), and the proportion of protected area was positively associated with incidence on duck farms (significant associations were identified in models 1, 2, 4, 5, and 6; the respective ORs were 1.027, 1.026, 1.018, 1.019, and 1.016). altitude was negatively associated with incidence on chicken farms (significant associations were identified in models 2–6; the respective ORs were 0.988, 0.993, 0.991, 0.994, and 0.991). Significant negative associations were detected for predator species richness on chicken and duck farms, particularly for models in which the study area included regions with reservoir suitability exceeding 20%–30%. Significant associations with predator species richness were identified in models 3–6 for chicken farms; the respective ORs were 0.601, 0.655, 0.598, and 0.645. Significant associations with predator species richness were identified in models 2–6 for duck farms; the respective ORs were 0.329, 0.317, 0.299, 0.351, and 0.401.

## 4 Discussion

The associations between environmental factors and the incidence of HPAI during the initial phase of the six outbreaks between 2010–2021 were investigated in this aggregate-level study. The results showed that temperature, altitude, and predator species richness were negatively associated, whereas farm density was positively associated, with the incidence of HPAI on chicken farms. Temperature and predator species richness were negatively associated, and the proportion of protected area was positively associated, with the incidence of HPAI on duck farms. Associations with mean humidity, mean precipitation, and deforestation level were not statistically significant on chicken or duck farms.

The distinct patterns of association according to poultry species suggest different transmission routes between chicken and duck farms. The significant positive association with farm density on chicken farms indicated that the incidence of HPAI on chicken farms could be attributed to transmission by previously infected farms. Higher density often increases contact between farms. In contrast, there was a significant positive association between the proportion of protected area and the incidence of HPAI on duck farms. Considering that the abundance of wild birds is generally higher in protected regions (31), these associations suggest that the major transmission route for duck farms could be spillover transmission from wild birds. This finding is consistent with descriptions in a previous epidemiological report (32), thus indicating that the incidence of HPAI on duck farms during the initial phase of an outbreak often occurs near wild bird habitats, implying that major exposure route of duck farms could be spillover from wild birds. Associations of altitude in chicken farms were generally negative, but there were no significant associations in duck farms. The significant association was consistent with previous studies in Southeast Asia, China and South Korea (33–35). In those studies, altitude was considered as proxy of unmeasured risk factors. In this study, interaction density between farms (vehicle movement between farms) was not included as an explanatory variable although it is highly related to transmission between farms. Considering that higher altitude can indicate mountainous area in South Korea, the lower altitude could indicate higher interaction. The null association in duck farms can be explained by hypothesis that the major cause of infection for duck farms is spillover from wild birds, although further studies are required to confirm.

The significant negative associations with mean temperature are consistent with the results of previous experimental studies. Paek et al. (36) showed that the survival of avian influenza virus tends to increase at lower temperatures. Public health studies in humans have also shown negative associations between temperature and the incidence of influenza (37). Conversely, meteorological factors (e.g., humidity and precipitation) were not significantly associated with the incidence of HPAI. These findings were inconsistent with previous studies (38, 39), which showed significant associations and plausible mechanisms regarding viral activities and wild bird ecology. Although further studies are recommended to explore this inconsistency, the lack of associations in the present study may be attributed to the small number of units included.

Predator species richness was negatively associated with the incidence of HPAI on chicken and duck farms. Although protective effects could not be confirmed in the present study, these results supported our hypothesis that higher predator species richness could mitigate activity in wild reservoirs (40), thereby decreasing the risk of spillover. The results of previous studies have suggested significant negative associations with predator species in various rodent-borne diseases (41, 42); to our knowledge, the present report is the first to demonstrate a significant association on the basis of HPAI data. This increasing evidence suggests that predatory species diversity has a protective effect, but further confirmatory studies with different diseases in various ecological contexts are needed. Previous studies have suggested that avian predator species can be infected by HPAI and thereby spread the virus to domestic poultry farms (43, 44). However, the infection for raptors can be lethal (45), and cross-species transmission from the raptors could be rare than wild duck species (46) which shows mild symptom but keep spreading the viruses.

The association with deforestation level was not statistically significant on chicken or duck farms. Although deforestation modifies wild bird migration, thereby increasing contact between avian populations and poultry farms (47), the current findings were not consistent with an effect of deforestation. The low statistical power from the small number of study units could have contributed to the lack of significant findings. The ecology of HPAI reservoirs could also explain this lack of association. Dominant reservoirs in South Korea, such as *A. poecilorhyncha*, generally prefer to inhabit bodies of water or agricultural lands, rather than mountainous regions (31). Therefore, the effects of deforestation on population-level changes in avian activity are limited.

The following limitations should be considered when interpreting the results of this study. First, some cases during the initial phase of outbreaks were attributed to horizontal transmission. Although definitive methodologies have not been established, plausible techniques to classify the source of infection (i.e., spillover from wild birds or horizontal transmission from infected farms) include phylogenetic and epidemiological investigations. Future studies should use a sophisticated approach to minimize misclassification. Second, the biosecurity levels of poultry farms were not included in the models. Because farm-level biosecurity rarely affects environmental drivers, biosecurity level was not a confounding factor. However, such information should be included in future studies. Third, the spatial resolution of predator species richness was limited. Because most predators are endangered species, it was not possible to obtain point-level occurrence data; only district-level data were utilized. Predatory species data with higher resolution could have produced different results. Fourth, some temporally dynamic variables such as deforestation and protected area were included as temporally fixed variables. Although spatial variations over time could be limited, it can reduce statistical power by increasing random error of the explanatory variables.

Nonetheless, the present study has effectively identified significant environmental drivers of the initial phase of HPAI outbreaks in South Korea. These findings hold considerable promise for future predictive research, as the identified variables can serve as robust predictors. Furthermore, the noteworthy correlation observed between predator species richness and HPAI outbreaks carries important implications from a conservation perspective. Given that many predator species in South Korea are currently endangered, significant conservation initiatives have been undertaken. Demonstrating the utility of predator species in disease control could potentially strengthen advocacy efforts for the conservation of these species.

## 5 Conclusion

In this study, we investigated environmental drivers for the incidence of HPAI on chicken and duck farms. The dominant transmission routes on chicken and duck farms were suspected to be horizontal and spillover, respectively; we also found that temperature, which influences viral activity, was negatively associated with the incidence of HPAI on both types of farms. To our knowledge, the present study is the first to reveal a potential protective effect of predator species richness against HPAI outbreaks. These findings can be used to develop prediction models for the initial phase of an HPAI outbreak; such models could support the implementation of a risk-based surveillance system.

## Data availability statement

Publicly available datasets were analyzed in this study. This data can be found at: https://www.dropbox.com/scl/fi/iwmejw3z6gudp4h86r2ps/dat_base_022022.csv?rlkey=4eszr366ju5krdmrw0h81l178&dl=0.

## Ethics statement

Ethical approval was not required for the study involving animals in accordance with the local legislation and institutional requirements because only secondary data that publicly available by Korean government has been used.

## Author contributions

K-DM: Conceptualization, Data curation, Formal analysis, Funding acquisition, Investigation, Methodology, Project administration, Resources, Software, Supervision, Validation, Visualization, Writing – original draft, Writing – review & editing. D-sY: Conceptualization, Supervision, Writing – original draft, Writing – review & editing.
