# Supplementary material for: Ecological drivers for poultry farms predisposed to highly pathogenic avian influenza virus infection during the initial phase of the six outbreaks between 2010–2021: a nationwide study in South Korea
Source: Front Vet Sci. 2023 Dec 7;10:1278852. doi: 10.3389/fvets.2023.1278852 (PMC10733472; doi:10.3389/fvets.2023.1278852)
Supplement: Supplementary file 1 [file Data_Sheet_1.docx]

**Supplementary Materials**

Table S1. Numbers of case and control regions for models 1–6

| Model | Chicken | | Duck | |
| --- | --- | --- | --- | --- |
|  | Case | Control | Case | Control |
| Model 1 | 14 | 385 | 20 | 38 |
| Model 2 | 19 | 380 | 22 | 36 |
| Model 3 | 43 | 766 | 34 | 65 |
| Model 4 | 64 | 745 | 40 | 59 |
| Model 5 | 52 | 1,165 | 34 | 99 |
| Model 6 | 86 | 1,131 | 42 | 91 |

Note: An ecological study design was implemented, and the unit of analysis was the neighborhood. The analysis was stratified according to animal species (chicken and duck farms).


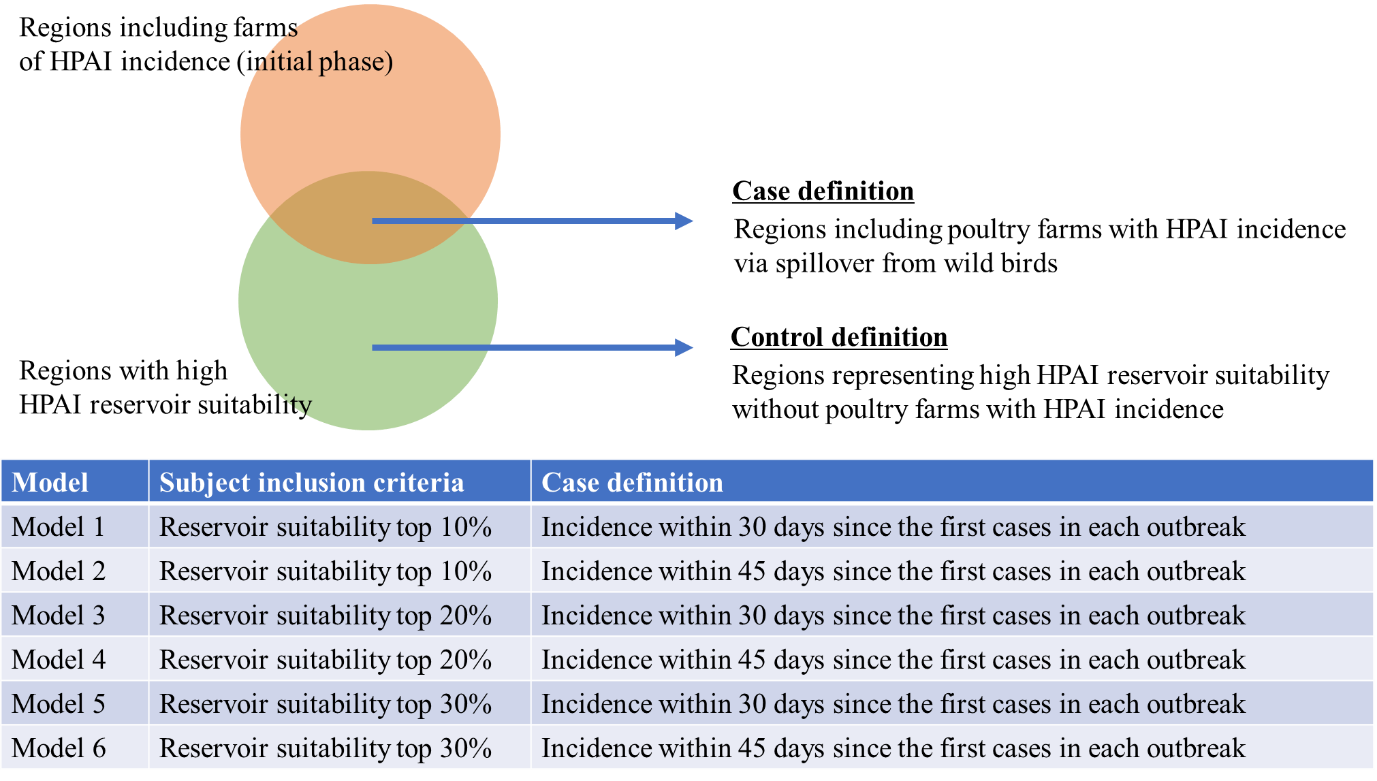


Figure S1. Definitions of case and control regions in this study.

Note: An ecological study design was implemented, and the unit of analysis was the neighborhood.


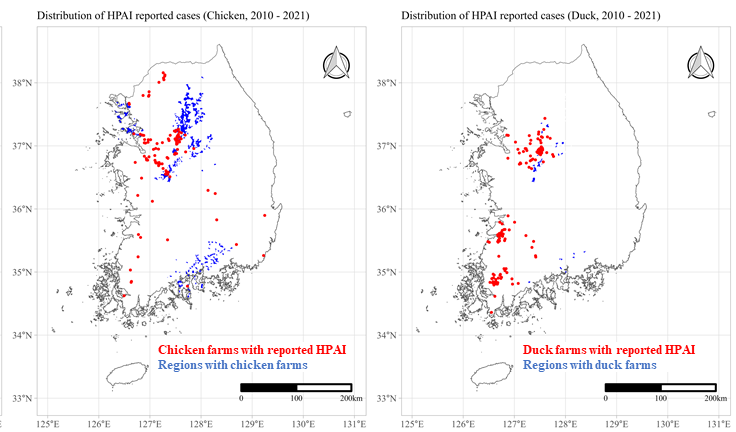


Figure S2. Locations of case and control regions for model 1

Note: The numbers of case and control regions for chicken farm model 1 were 14 and 385, respectively (left). The numbers of case and control regions for duck farm model 1 were 20 and 38, respectively (right).


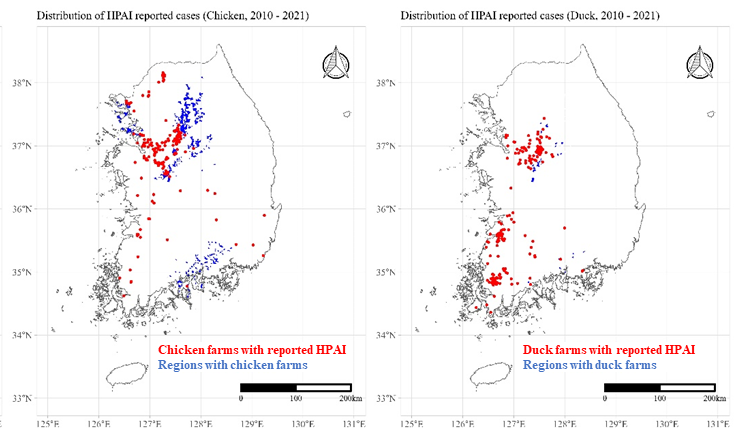


Figure S3. Locations of case and control regions for model 2

Note: The numbers of case and control regions for chicken farm model 2 were 19 and 380, respectively (left). The numbers of case and control regions for duck farm model 2 were 22 and 36, respectively (right).


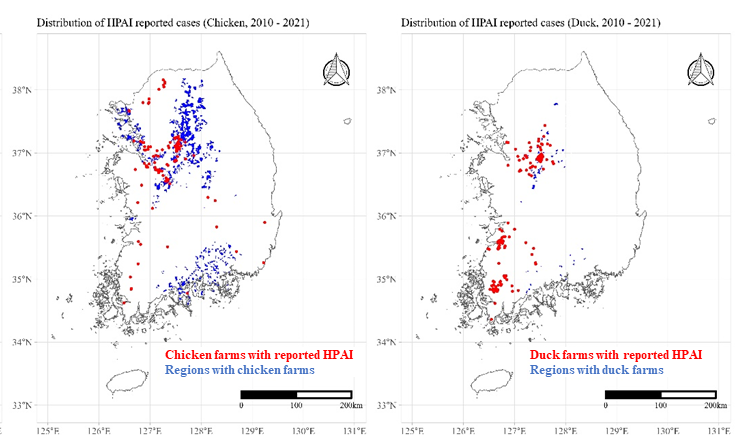


Figure S4. Locations of case and control regions for model 3

Note: The numbers of case and control regions for chicken farm model 3 were 43 and 766, respectively (left). The numbers of case and control regions for duck farm model 3 were 34 and 65, respectively (right).


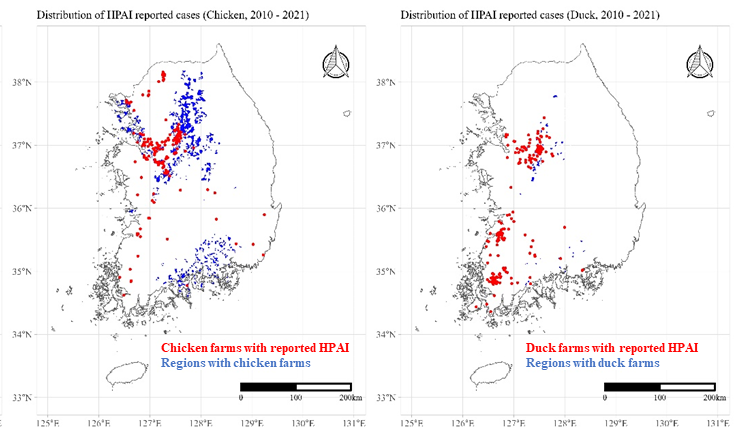


Figure S5. Locations of case and control regions for model 4

Note: The numbers of case and control regions for chicken farm model 4 were 64 and 745, respectively (left). The numbers of case and control regions for duck farm model 4 were 40 and 59, respectively (right).


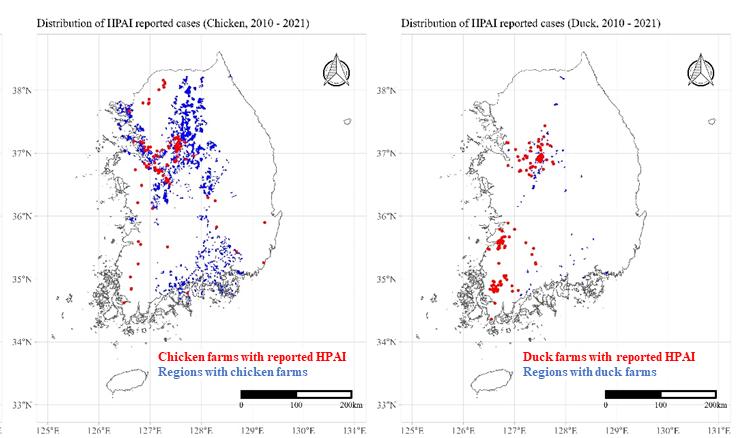


Figure S6. Locations of case and control regions for model 5

Note: The numbers of case and control regions for chicken farm model 5 were 52 and 1,165, respectively (left). The numbers of case and control regions for duck farm model 5 were 34 and 99, respectively (right).


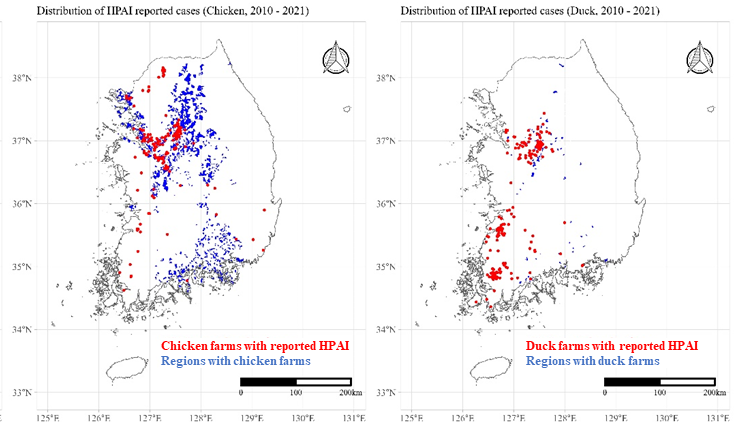


Figure S7. Locations of case and control regions for model 6

Note: The numbers of case and control regions for chicken farm model 6 were 86 and 1,131, respectively (left). The numbers of case and control regions for duck farm model 6 were 42 and 91, respectively (right).


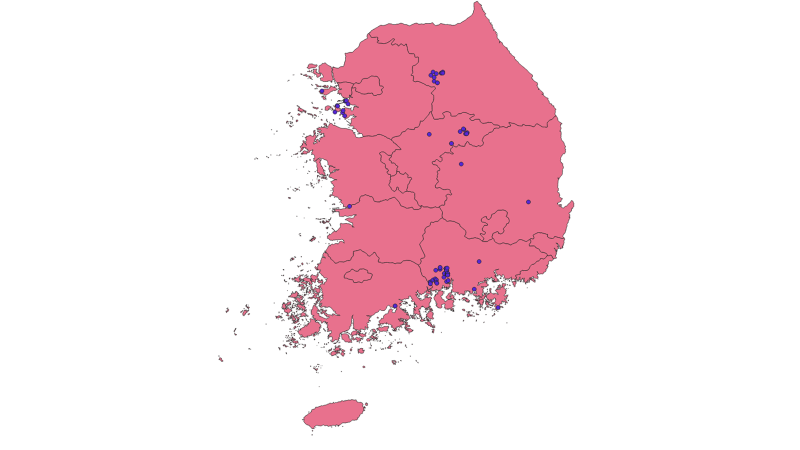


Figure S8. Locations of reported *Anas platyrhynchos* occurrence according to the National Ecosystem Survey
